# Radiation myelitis after hypofractionated radiotherapy with concomitant gefitinib

**DOI:** 10.1186/s13014-015-0334-7

**Published:** 2015-01-29

**Authors:** Victor Lewitzki, Nicolaus Andratschke, Thomas Kuhnt, Guido Hildebrandt

**Affiliations:** Department of Radiation Oncology, University Medicine Rostock, Südring 75, 18059 Rostock, Germany; Department of Radiation Oncology, University Zürich, Rämistrasse 100, 8091 Zürich, Switzerland; Department of Radiation Oncology, University Leipzig, Stephanstraße 9a, 04103 Leipzig, Germany; Department of Radiation Oncology, University Würzburg, Joseph Schneider Str. 11, 97080 Würzburg, Germany

**Keywords:** Radiation myelitis, Concomitant radiotherapy, Gefitinib

## Abstract

We describe the case of a 71-year-old Caucasian female with primary disseminated non-small cell cancer of the lung, presented for palliative radiotherapy of metastatic spread to the 9th and 11th thoracic vertebrae without intramedullary growth. Palliative radiotherapy with daily fractions of 3 Gy and a cumulative dose of 36 Gy to thoracic vertebrae 8-12 was performed. The patient received concomitantly 250 mg gefitinib daily. After a latent period of 16 months, the patient developed symptoms of myelitis. Magnetic resonance imaging (MRI) did not reveal any bony or intraspinal tumor progression, but spinal cord signal alteration. No response to steroids was achieved. The neurological symptoms were progressive in August 2013 with the right leg being completely plegic. The left leg was incompletely paralyzed. Deep and superficial sensitivity was also diminished bilaterally. The patient was completely urinary and anally incontinent. Contrary to the clinical findings, a follow-up MRI (July 2013) showed amelioration of the former signal alterations in the spinal cord. The diagnosis of paraneoplastic myelopathy was refuted by a negative test for autologous antibodies. At the last clinical visit in May 2014, the neurological symptoms were stable. The last tumor-specific treatment the patient is receiving is erlotinib 125 mg/d.

We reviewed the literature and found no reported cases of radiation myelopathy after the treatment in such a setting. The calculated probability of such complication after radiotherapy alone is statistically measurable at the level of 0.02%. We suppose that gefitinib could also play a role in the development of this rare complication.

## Background

Radiation myelitis [RM] is a rare condition, since modern treatment techniques with a homogeneous dose distribution are available and critical doses for human spine are well known. Thus a moderate hypofractionated radiotherapy of the thoracic spine with 36 Gy in 12 daily fractions of 3 Gy is believed to be safe. A possible interaction of spinal cord irradiation with gefitinib is not known. Radiobiological experiments indicate a possible interaction with gefitinib in some selected cancer cell lines. No data on normal tissue tolerance of this combination are available.

Lung cancer is a common malignant disease, often with distant metastases as first presenting sign of malignant disease. The common site of spread is the skeleton, with symptomatic vertebral spread sometimes causing the first symptoms of this disease. Along with palliative chemotherapy and bone modifying agents, radiotherapy plays an important role in the palliation of symptomatic bone metastases. Due to progress in treatment planning and delivery, allowing for a better dose distribution and homogenous dose application, and growing experience in the tolerance of human spinal cord to irradiation, radiation myelitis is fortunately a rare but severe complication of irradiation.

A hypofractionated treatment schedule is a common option for radiotherapy in a palliative setting. The main goal of such a treatment is good symptom control with short treatment time. A total dose of 36 Gy in 12 daily fractions of 3 Gy is a possible fractionation schedule for the treatment of bone metastases. One meta-analysis of three randomized studies in patients with non-small cell lung cancer (NSCLC) revealed no cases of radiation myelitis in 86 patients treated by this modality [1].

Gefitinib is a selective inhibitor of the tyrosine kinase. It is approved for primary treatment of metastatic lung adenocarcinoma with epidermal growth factor receptor (EGFR) gene mutation. The low rate of adverse events and often prolonged response make its use very attractive especially in the palliative situation [2]. There are several trials and retrospective series also demonstrating its activity in disease metastatic to the central nervous system. Dose dependence in achieving objective and clinical response was reported [3]. Findings from these studies are at least suggestive of interactions between ionizing radiation and gefitinib [4,5]. There are also radiobiological studies to support an interaction of gefitinib in at least some cell lines [6-8].

To date, we have found just one reported case of radiation myelitis after concomitant definitive high-dose radiation and chemotherapy including gefitinib [9].

In this article we present a case of radiation myelopathy after palliative radiotherapy applying a dose of 36 Gy in 12 fractions to the 8-12 thoracic vertebrae concomitant with 250 mg/m^2^ gefitinib daily.

### Case report

In June 2011, a 70-year-old female Caucasian patient presented with weight loss and hematuria in a local hospital. Further diagnostics revealed lung tumor in the left lobe with multiple pulmonary, liver, left adrenal gland and bone metastases, including pathological fracture of the 11th vertebra. The tumor cytology showed adenocarcinoma. Thus, palliative chemotherapy with carboplatin/paclitaxel was administered. A further genetic analysis of a tumor biopsy proved EGFR mutation at exon 19. After another hospital attendance due to urinary infection with complications, the chemotherapy was stopped and targeted therapy with gefitinib at a dose of 250 mg/m^2^ was started on August 27, 2011. In September 2011, a palliative course of radiotherapy with daily single doses of 3 Gy to a total dose of 36 Gy was administered to vertebrae 8-12 for symptomatic metastatic spread to thoracic vertebrae 9 and 11 without extraosseous and intraspinal tumor manifestations. Daily concomitant administration of gefitinib was continued. On the last day of radiotherapy, the patient was reported to be completely free of metastasis-related symptoms.

In December 2011, restaging proved partial response of primary tumor and lung metastases with complete response of liver and adrenal metastases. Maintenance therapy with gefitinib was continued. In March 2012, the patient presented with back pain approximately at level Th 6-7. Restaging with thoracic and abdominal computed tomography in April 2012 showed stable disease as compared to December 2011. In August 2012, the bisphosphonate therapy was stopped due to no otherwise specified adverse events and was switched to denosumab. Due to progressive disease revealed in September 2012, systemic therapy was changed to pemetrexed and continued after achieving stable disease in December 2012.

**Figure 1:**
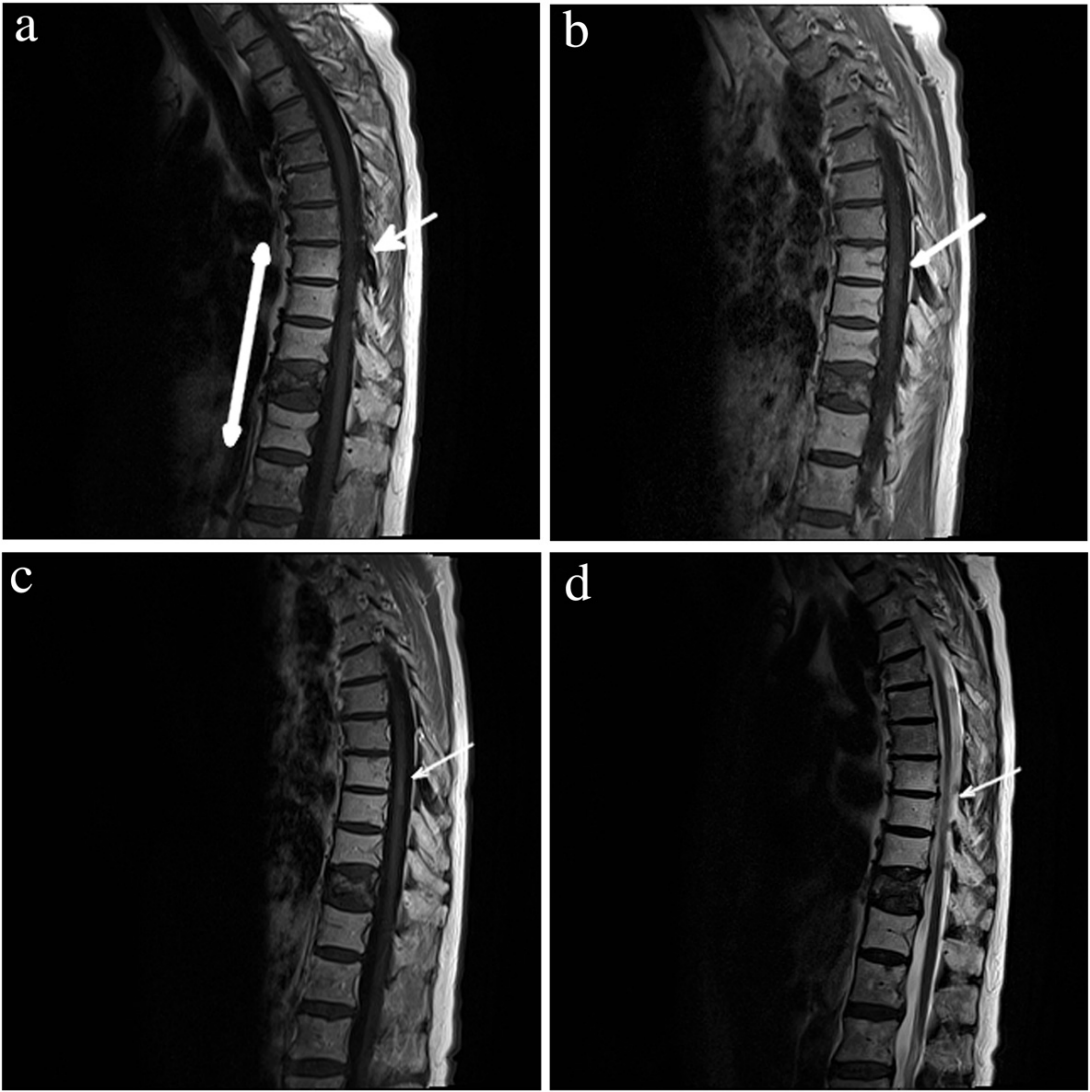
**Thoracic cord magnetic resonance imaging of 71 year old patient in the course of radiation myelitis. a)** February 2013: T1-weighted MRI scan without contrast shows hyperintense post-actinic changes in vertebrae Th 8-12, isointense changes in segments Th 7-9 and Th 11 fracture. **b)** February 2013: T1-weighted MRI with gadolinium demonstrates a ring-shaped posterior enhancement in spinal cord (arrow) at the level of Th 8-9. **c)** July 2013: T1-weighted MRI with gadolinium demonstrates a decrease of the ring-shaped posterior enhancement in spinal cord (arrow) at the level of Th 8-9 in comparison with the previous study (Figure 1b). **d)** July 2013: T2-weighted MRI scans demonstrate no signs of edema.

16 months after radiotherapy (January 2013), the patient presented with pain in her right hip radiating down to the lower leg. Spinal computed tomography in February 2013 did not reveal any progression of bony spinal metastases. The patient did not respond to analgesic therapy with Tapentadol 3×100 mg/die. Due to neuropathic complaints in the right leg and muscular weakness, the patient was hospitalized to the Neurological department of the regional clinic in February 2013. Spinal MRI with gadolinium contrast agent showed T2-hypertintense spinal cord lesions in segment Th 7-10 with a little contrast enhancement on T1 and isointense in native T1 (Figure 1a, b). Cranial MRI revealed one small cerebellar lesion suspected to be of metastatic origin. Spinal fluid taken was negative for tumor cells. On neurological examination, pyramid signs were negative. The muscle tonus, and the deep and superficial sensitivity of the right leg were diminished. Electromyography of right m. tibialis anterior and m. rectus femoris showed no pathological spontaneous activity. Controlled muscular activity was sluggish.

The patient was consulted in our Radiological department. We consulted with our Radiologist on the MRIs and discussed differential diagnoses. A diagnosis of radiation-induced myelopathy was suspected and dexamethasone was proposed as a therapy for myelitis. The patient did not respond to intravenous dexamethasone 24 mg/die. A few weeks later, she became urinary incontinent. Some pain relief could be obtained with pregabalin 75 mg bid. The patient continued her palliative chemotherapy in an outpatient setting.

The neurological symptoms were progressive in August 2013 with the right leg being completely plegic. The left leg was incompletely paralyzed. Deep sensitivity was completely absent in the whole right leg and up to the knee on the left side. Superficial sensitivity was also diminished in accordance with deep sensitivity bilaterally. The patient was completely urinary and anally incontinent. Contrary to the clinical findings, a follow-up MRI (July 2013) showed amelioration of the former signal alterations in the spinal cord (Figure 1c, d). The diagnosis of paraneoplastic myelopathy was refuted by a negative test for autologous antibodies performed in our institution.

At the last visit (May 2014), the neurological symptoms were stable in comparison with the state in August 2013. At the last restaging (April 2014) of the thoracic CT, the primary tumor was slightly progredient. No intraspinal changes were described in the thoracic CT. The current targeted therapy contains erlotinib in the highest tolerable dosage for this patient of 125 mg daily. Before the last progression, the patient received erlotinib 100 mg daily with diarrhea being a dose-limiting toxicity.

## Discussion

In recent decades, radiation myelitis has become a rare complication, reflecting the confidence and expertise in dose prescription and delivery due to sophisticated techniques and clinical expertise of spinal cord tolerance. In the current literature, one can find more discussion on the problems of radiosurgery with high single dose irradiation or re-irradiation of spinal cord or adherent structures, rather than clinical findings of radiation myelitis following conventional radiotherapy [10]. On the other hand, the rapid progress in drug development, with a plethora of novel targeted drugs and a lack of basic radiobiological data on their combination with radiotherapy, has left us with questions about possible interactions each time, when such toxicity follows our well known “thought to be safe” therapeutic regimen. Nevertheless, long-lasting responses with these new substances can be achieved in some patients, thus giving our patients the chance of longer survival and the development of complications rarely before seen due to previously limited survival rates.

The diagnosis in our case was supported by the typical clinical picture with symptom onset at 16 months after radiotherapy with progressive sensory and motor loss, urinary incontinence and typical MRI presentation, consistent with well-known data [11,12], with a posteriorly located ring-shaped contrast-enhanced lesion at the irradiated level and extensive edema in the upper part outside the treatment field. Neurological findings were also consistent with RM diagnosis. The progressive clinical neurological deficits with the corresponding radiological findings without evidence of progressive metastatic lesions are typical of the development of RM refuting intraspinal metastasis. Finally, in the absence of auto-antibodies (aminophysin, CV2, PNMa2, Ri, Anti-Yo, Hu) the diagnosis of paraneoplastic myelopathy could be withdrawn. Thus, not having a histological confirmation, we are confident of the diagnosis of RM in the case described. The treatment with steroids, having a limited influence on the development of this complication, cannot assist in proving or withdrawing the diagnosis of RM.

Using the linear-quadratic model generally adopted to compare a dose normalized to conventional fractionation (EQD_2_) (${\mathrm{EQD}}_2=D\ast \frac{d+\left(\alpha \beta \right)}{2 Gy+\left(\alpha \beta \right)}$ with α/β = 3 Gy, D = cumulative dose and d = dose per fraction), the doses applied to the patient equal an EQD_2_ of 43.2 Gy. Even after applying an α/β of 2 Gy an EQD_2_ of 45 Gy is still considered to be safe. Using the data of Schultheiss [13], evaluating the radiation dose response for cervical spinal cord to be more sensitive than thoracic [14], we can assume that a probability of myelopathy at EQD_2_ 45 Gy is lower than 0.03 percent.

Using data of the QUANTEC review [15], we could suggest a probability of 0.2 percent of myelopathy after conventional fractionated radiation with 50 Gy in 2 Gy single fractions, but not after the hypofractionated radiation being applied in this case.

Dunst *et al.* reported one case of radiation myelitis after application of a total of 25 Gy in 5 daily fractions of 5 Gy, assuming a maximal dose at spinal cord of 27 Gy, (EQD_2_ 49.95 Gy with α/β =2) [16]. Also looking at this case of above-average radiation sensitivity, it should be mentioned that EQD_2_ in the case reported by Dunst *et al.* is 4.95 Gy higher than in our patient.

Macbeth *et al.* reported 5 cases of radiation myelopathy among 1048 patients with inoperable non-small cell lung cancer, treated with palliative radiotherapy in three randomized trials conducted by the Medical Research Council Lung Cancer Working Party. Of the 5 instances of radiation myelopathy, 3 occurred in the 524 patients treated with 17 Gy in 2 fractions, and 2 in the 153 patients treated with 39 Gy in 13 fractions. There was also a group of 86 patients treated with 36 Gy in 12 fractions. The annual risks in this study had wide 95% confidence intervals. According to this study, the authors are suggesting that α/β is possibly close to 2 Gy [1].

Matha *et al.* described a case of a 47-year-old male who developed a cervical myelopathy after radiochemotherapy of tongue cancer. For this patient a total dose of 70 Gy over 7 weeks concurrently with weekly cisplatin of 40 mg/m^2^ was applied. The maximum dose in the spinal cord was limited to 44.8 Gy. The first symptom, developing in this patient approximately 7 months after radiotherapy, was sensory loss bilaterally from the nipples down. His symptoms worsened and he also developed bowel and bladder incontinence, gait instability and mild weakness of all four extremities. He had diminished sensation to light touch, pinprick, and temperature from C4 down. Magnetic resonance imaging of his spinal cord revealed a bright lesion at T2, accumulating gadolinium with corresponding enhancement at T1 [17]. This patient died consequently on the sequela of RM.

Considering possible limitations of radiobiological models, we want to emphasize that empiric data from previous studies, reporting no cases of radiation myelitis with this fractionation schedule, do not exclude the possibility of radiation myelitis after a cumulative dose of 36 Gy in 12 daily fractions. There are also some limitations using older data from 2-D planned radiotherapy, due to relatively imprecise dosimetry and inhomogeneous dose distribution with hot spots regularly presented inside target volumes.

Discussing the possible influence of gefitinib on the radiosensitivity of spinal cord, one should consider that experimental data addressing this question do not exist. There are two widely accepted means of organ damage in the pathogenesis of RM: damage of glial cells (glial theory) and damage of vascular endothelial cells (vascular hypothesis). The latter seems to be better proven by experimental data and observations, in that the vascular architecture of the spinal cord can often be aligned with pathological patterns of myelitis [18]. There are also experimental [19,20] and clinical data [21,22] published, indirectly supporting the vascular hypothesis. Gefitinib seems to suppress mobilization of pericytes needed for vessel stabilization [20], at least in tumor models.

There are clinical data from phase I and III studies, showing a higher occurrence of tumor hemorrhage under treatment with gefitinib concurrent to IR [22,9]. One out of 23 patients treated with radiation and concurrent carboplatin, paclitaxel and gefitinib, developed grade 3 anterior spinal cord syndrome. Spinal cord infarct within the radiation field was the probable reason. The clinical changes started 8 months after completion of radiation and 2 weeks after discontinuing gefitinib. The symptoms were lower extremity weakness and tingling for 1 to 2 months and new onset of urinary retention. The total maximal calculated dose in spinal cord was 49.6 Gy. The patient was evaluated with magnetic resonance imaging of the spine, which was negative for cord compression. A lumbar puncture was negative for malignant cells [9].

Experimental data, answering the same question in normal tissues irradiated, especially in mammalian spinal cord, are needed to answer this specific question.

Until these data are available, we suggest paying special attention when using concurrent radiotherapy with gefitinib, in particular with spinal cord being a part of the target volume and if the cervical or thoracic spine is involved.

## Conclusion

Radiotherapy with a cumulative dose of 36 Gy in 12 daily fractions of 3 Gy is believed to be a safe option for the palliative treatment of vertebral metastasis. Myelitis is a rare complication at this dose level. There are no data about this complication as a sequela of concomitant radiotherapy to 36 Gy with gefitinib. Until more clinical data are available, we suggest that a combination of radiotherapy and gefitinib should be used with caution regarding their possible interaction in tissue reactions of the spinal cord.

We also suggest being especially cautious in choosing the modality of palliative radiotherapy for patients with NSCLC with known EGFR mutation. Due to the targeted therapies, some of these patients can achieve very long survival thus having time to develop late sequelae of radiation therapy.

### Consent

The patient has given her written consent for the case report to be published.
